# Clinical Evaluation of Rectus Sheath Block Versus Local Anesthetic Infiltration in Laparoscopic Inguinal Hernia Repair: A Retrospective Comparative Study

**DOI:** 10.7759/cureus.98039

**Published:** 2025-11-28

**Authors:** Usama A Abdelgwad, Khaled Abuamra, Ateeq Ahmed, Michael A Rizk, Aicha A Idrissi, Julio César Padrón La Rosa, Sherin Abdelhamid

**Affiliations:** 1 Department of Anesthesia, Dubai Hospital, Dubai Health, Dubai, ARE

**Keywords:** dexmedetomidine, laparoscopic inguinal hernia repair, local anesthetic infiltration, opioid-sparing anesthesia, postoperative analgesia, rectus sheath block

## Abstract

Background

Laparoscopic inguinal hernia repair is frequently associated with early postoperative pain due to somatic nociceptive input from the anterior abdominal wall. Ultrasound-guided rectus sheath block (RSB) provides targeted analgesia by blocking terminal branches of the thoracoabdominal nerves within the rectus sheath. Although RSB is increasingly used in minimally invasive surgery, its comparative effectiveness against conventional local anesthetic (LA) wound infiltration in adult hernia repair remains inadequately characterized.

Objectives

The objective of this study is to evaluate the clinical effect of pre-incisional RSB versus postoperative LA infiltration on intraoperative opioid and dexmedetomidine requirements, as well as postoperative analgesic use, in adults undergoing laparoscopic total extraperitoneal (TEP) inguinal hernia repair.

Methods

This retrospective cohort study included 211 adult patients who underwent laparoscopic TEP inguinal hernia repair at Dubai Hospital between 2018 and 2025. Patients received either ultrasound-guided RSB before skin incision (n = 102) or surgeon-administered LA infiltration at the end of surgery (n = 109). Data were extracted from electronic anesthesia records and included demographics, ASA classification, body mass index (BMI), surgical duration, laterality, and intra-/postoperative analgesic consumption. Statistical analyses employed Mann-Whitney U, chi-square, and multivariable linear regression tests to adjust for surgical duration and laterality. The primary outcome was intraoperative opioid dose (morphine-equivalent mg). Secondary outcomes included intraoperative dexmedetomidine rate (µg/kg/h) and postoperative opioid and non-opioid analgesic use.

Results

Baseline demographic variables were comparable between groups, though operative duration and bilateral repairs were higher in the RSB group (p < 0.01). Pre-incisional RSB was associated with a significant reduction in intraoperative opioid requirement (median 3 mg vs 6 mg morphine-equivalent; p < 0.001). Regression analysis confirmed RSB as an independent predictor of lower opioid consumption (adjusted β = -3.53 mg; 95% CI -4.63 to -2.42; p < 0.0001). The median intraoperative dexmedetomidine infusion rate was lower with RSB (0.227 µg/kg/h (IQR 0.184)) versus LA (0.327 µg/kg/h (IQR 0.327); p = 0.021), but the difference lost statistical significance after adjustment (adjusted p = 0.194). Postoperative opioid requirements were minimal in both groups (median 0 mg), while RSB patients required significantly fewer non-opioid analgesic doses (paracetamol/NSAIDs, p < 0.05) and exhibited a higher incidence of complete analgesia avoidance (19.6% vs 8.3%).

Conclusions

Pre-incisional ultrasound-guided RSB independently reduces intraoperative opioid exposure compared with LA wound infiltration in laparoscopic TEP inguinal hernia repair. Although dexmedetomidine sparing was not significant after adjustment, the overall multimodal analgesic burden was lower in the RSB group. These findings support RSB as an effective, safe, and opioid-sparing regional technique for adult laparoscopic hernia repair.

## Introduction

The rectus sheath block (RSB) is a regional anesthetic technique commonly used for midline abdominal surgeries, particularly those involving infraumbilical incisions. It works by delivering a local anesthetic (LA) between the rectus abdominis muscle and its posterior sheath, targeting the anterior branches of the lower thoracic intercostal nerves (T7-T12) that innervate the central anterior abdominal wall [1,2]. RSB is valued for its ability to reduce somatic pain and minimize opioid requirements in procedures involving midline incisions [3].

Laparoscopic inguinal hernia repair, whether performed via the totally extraperitoneal (TEP) technique, involves manipulation of both midline and lateral abdominal structures. Trocar insertion typically occurs near the umbilicus (midline), while mesh placement and peritoneal dissection extend to the inguinal region (lateral) [4,5]. This dual distribution of surgical trauma produces a combination of somatic and potentially visceral pain, making postoperative analgesia more complex than in purely midline procedures [6].

Despite the widespread use of RSB in midline surgeries, limited evidence exists regarding its effectiveness as a sole analgesic technique for laparoscopic inguinal hernia repair. Most existing literature focuses on pediatric populations [7] or the use of RSB in combination with other techniques such as transversus abdominis plane (TAP) blocks or systemic analgesics [8]. There remains a significant knowledge gap on whether RSB alone provides adequate coverage for the full pain profile associated with TEP repairs in adults.

This retrospective comparative study aims to evaluate the effectiveness of the RSB as a sole regional analgesic technique compared with conventional local anesthetic infiltration, which remains the most commonly practiced method for laparoscopic inguinal hernia repair. By analyzing intraoperative opioid consumption, intraoperative dexmedetomidine use as a sedative and non-opioid analgesic adjuvant, and postoperative opioid and non-opioid analgesic requirements, this study provides real-world evidence to inform perioperative pain management strategies in minimally invasive hernia surgery.

## Materials and methods

This retrospective, single-center, comparative observational study was conducted at Dubai Hospital, Dubai Health, United Arab Emirates. The study included adult patients who underwent elective laparoscopic inguinal hernia repair under general anesthesia between January 2018 and April 2025. Two perioperative analgesic strategies were compared: pre-incisional ultrasound-guided RSB and surgeon-administered LA infiltration at wound closure. Ethical approval was obtained from the Dubai Health Authority Human Research Ethics Committee (approval number DSREC-05/2025_13).

Study population

All adults aged ≥ 18 years who underwent transabdominal preperitoneal TEP laparoscopic inguinal hernia repair were eligible. Patients were grouped according to the analgesic technique recorded in the anesthesia chart: Group RSB received a pre-incisional ultrasound-guided rectus sheath block, and Group LA received LA infiltration at skin closure. A total-population sampling approach was applied, including all eligible cases during the study period. No formal a priori sample-size calculation was performed because of the retrospective design; all eligible cases were included to maximize precision. Effect sizes with 95% confidence intervals (CIs) are reported.

Inclusion and exclusion criteria

Included patients were ASA physical status I-III adults undergoing elective laparoscopic hernia repair as a single procedure with either RSB or LA infiltration as the sole regional technique. Exclusion criteria were ASA IV or higher, emergency or converted-open cases, concurrent procedures, receipt of both techniques or other regional blocks (e.g., TAP, epidural, spinal), chronic opioid therapy within three months preoperatively, and incomplete medical records.

Anesthetic management

All patients were anesthetized by the same anesthesia team and operated on by the same surgical consultant to ensure consistency in perioperative management. As per the institutional protocol, general anesthesia was induced with fentanyl 1-2 µg/kg, propofol 2-4 mg/kg, and rocuronium 0.6-1 mg/kg to facilitate endotracheal intubation. Anesthesia was maintained with a volatile agent (sevoflurane or desflurane) in an oxygen/air mixture combined with a propofol infusion (50-150 µg/kg/min) as required for depth titration. Intraoperative analgesia was titrated to hemodynamic responses using incremental doses of opioids, supplemented with paracetamol 15 mg/kg, parecoxib 0.1 mg/kg, and dexamethasone 0.1 mg/kg administered intravenously after induction. Dexmedetomidine was given as an initial bolus of 0.5 µg/kg, followed by a variable infusion rate (0.2-0.7 µg/kg/h) adjusted according to clinical need and hemodynamic tolerance.

In the RSB group, the RSB was performed by the attending anesthesiologist after induction and before surgical incision, under ultrasound guidance using a high-frequency linear transducer (6-13 MHz) with a SonoSite X-Porte ultrasound system (FUJIFILM SonoSite Inc., Bothell, WA, USA) under strict aseptic precautions. The rectus sheath and posterior rectus sheath were visualized in the transverse plane, and local anesthetic was deposited between them using an in-plane lateral-to-medial approach. Local anesthetic (0.375% ropivacaine) was injected bilaterally between the posterior rectus sheath and rectus muscle along its lateral-posterior border, with a total volume of 40-60 mL depending on patient's body habitus. In the LA group, the surgeon infiltrated 0.375% ropivacaine at the trocar insertion sites, using a total volume of 15-20 mL. The anesthetic was infiltrated subcutaneously and administered at the end of surgery according to the operating surgeon’s standard practice. All patients were monitored continuously throughout the perioperative period using standard ASA monitoring.

Postoperative analgesia was standardized for all patients. Paracetamol 15 mg/kg (maximum 1 g) was administered three times daily as needed, combined with parecoxib 40 mg twice daily (as required) for VAS pain scores 1-6/10. Rescue opioid analgesia (intravenous morphine 2-3 mg increments) was administered only if the VAS score was≥ 7/10. This regimen was applied uniformly in both groups.

Data sources and variables

Data were retrieved from the electronic medical record system, operative notes, anesthesia charts, and nursing documentation. Extracted variables included demographic data (age, sex, BMI, ASA class), surgical characteristics (procedure type, laterality, duration), anesthetic details (technique, local-anesthetic use), intraoperative parameters (opioid consumption converted to morphine equivalents, dexmedetomidine infusion rate), and postoperative outcomes (pain scores at PACU, 6 h, 24 h; postoperative opioid/non-opioid use; rescue analgesia). All entries were verified and stored in a password-protected database accessible only to investigators.

To ensure uniform quantification of analgesic exposure, all intravenously administered opioids were standardized to intravenous morphine equivalents (IV-MEQ) using established equianalgesic conversion factors: oxycodone 1 mg = 1 mg IV morphine; nalbuphine 10 mg = 1 mg IV morphine; tramadol 10 mg = 1 mg IV morphine; and pethidine (meperidine) 10 mg = 1 mg IV morphine, with IV morphine serving as the reference standard.

Although VAS pain scores were available in the electronic medical record, they were frequently documented after analgesic administration, particularly by nursing staff. As a result, VAS values often reflected post-treatment states rather than untreated pain levels. Therefore, for consistency and accuracy, our analysis focused on the type and frequency of analgesic medications administered within the first 24 hours postoperatively. Analgesia was administered based on a standardized protocol: opioids were reserved for severe pain (VAS >7), while paracetamol and NSAIDs were given for mild-to-moderate pain (VAS 1-6). Medication administration was thus used as a surrogate marker for pain intensity. The primary outcome was total intraoperative and postoperative opioid requirement expressed as intravenous morphine-equivalent dose (mg). Secondary outcomes were intraoperative dexmedetomidine infusion rate, postoperative rescue analgesia, and postoperative non opioid analgesic use.

Statistical analysis

Data analysis was performed using IBM SPSS Statistics for Windows, Version 26 (Released 2019; IBM Corp., Armonk, New York, United States). Continuous variables were tested for normality using the Shapiro-Wilk test and are presented as mean ± standard deviation or median (interquartile range) as appropriate; categorical variables are expressed as frequencies and percentages. Between-group comparisons used the independent-samples t-test or Mann-Whitney U test for continuous data and the chi-square or Fisher’s exact test for categorical data. Multivariate linear and logistic regression models were applied to adjust for potential confounders such as age, ASA class, laterality, and operative duration. A p-value < 0.05 was considered statistically significant.

## Results

A total of 211 adult patients were included in the final analysis, comprising 102 patients who received RSB and 109 patients who received LA infiltration. Baseline demographic characteristics, including age, body mass index (BMI), sex distribution, and ASA physical status classification, were comparable between the two groups with no statistically significant differences (Table 1). However, significant differences were observed in operative parameters. The mean duration of surgery was significantly longer in the RSB group compared to the LA group (100.2 ± 31.4 vs. 85.3 ± 28.8 minutes, respectively; p < 0.001). Additionally, bilateral hernia repairs were more frequently performed in the RSB group (70.6%) than in the LA group (48.6%) (p = 0.002). These findings indicate potential baseline imbalances in surgical complexity and laterality that necessitated adjustment in subsequent regression analyses.

**Table 1:** Baseline Demographic and Operative Characteristics of Patients Receiving Rectus Sheath Block (RSB) Versus Local Anesthetic (LA) Infiltration. Data are presented as mean ± standard deviation (SD) for continuous variables and n (percentage) for categorical variables. Statistical significance was defined as p < 0.05. p-values for continuous variables were calculated using the independent samples t-test, and the corresponding t-values are reported. p-values for categorical variables were calculated using the Chi-square test, and the corresponding χ² (chi-square) values are reported.

Variable	RSB (n = 102)	LA (n = 109)	Test Statistic	p-value	Statistical Test
Age (years), Mean ± SD	55.3 ± 14.1	52.4 ± 16.3	t = 1.38	0.168	Independent t-test
BMI (kg/m²), Mean ± SD	27.4 ± 5.2	26.2 ± 3.8	t = 1.90	0.059	Independent t-test
Male sex, n (%)	99 (97.1%)	104 (95.4%)	χ² = 0.07	0.791	Chi-square test
ASA Physical Status, n (%)			χ² = 0.65	0.722	Chi-square test
ASA I	21 (20.6%)	27 (24.8%)			
ASA II	66 (64.7%)	65 (59.6%)			
ASA III	15 (14.7%)	17 (15.6%)			
Duration of surgery (min), Mean ± SD	100.2 ± 31.4	85.3 ± 28.8	t = 3.58	<0.001	Independent t-test
Laterality, n (%)			χ² = 9.64	0.002	Chi-square test
Bilateral	72 (70.6%)	53 (48.6%)			
Unilateral	30 (29.4%)	56 (51.4%)			

Intraoperative opioid consumption

**Figure 1:**
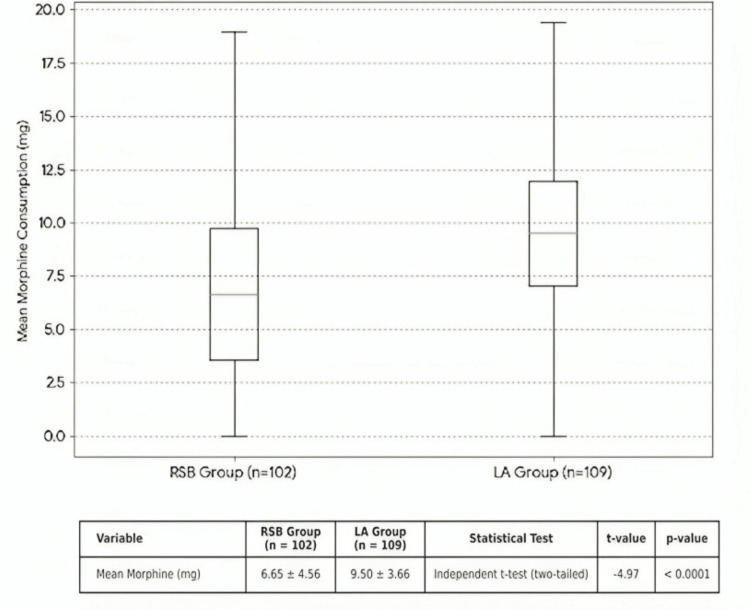
Unadjusted Distribution of Intraoperative Morphine Equivalent Consumption in Rectus Sheath Block (RSB) and the Local Anesthetic (LA) Infiltration Groups. Mean morphine consumption is shown as mean ± SD for the RSB group (n = 102) and LA group (n = 109). Independent t-test: t = −4.97, p < 0.0001 (significance threshold p < 0.05).

In terms of intraoperative opioid consumption, the mean converted morphine equivalent dose was significantly lower in the RSB group (6.65 mg) compared to the LA group (9.50 mg), with an independent samples t-test confirming this difference (p < 0.0001) (Figure 1).

**Figure 2:**
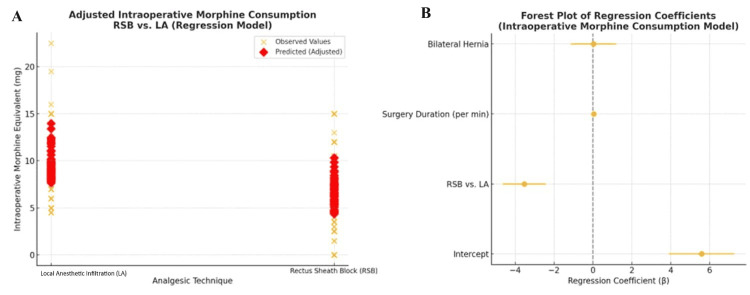
Adjusted Analysis of Intraoperative Morphine Consumption in Rectus Sheath Block (RSB) and the Local Anesthetic (LA) Infiltration Groups. (A) Observed and regression-adjusted intraoperative morphine equivalent consumption (mg) in patients receiving local anesthetic (LA) infiltration versus rectus sheath block (RSB). Orange X symbols represent observed values; red diamonds indicate adjusted predicted values from the multivariable linear regression model. (B) Forest plot of regression coefficients (β) from the model, including 95% confidence intervals. RSB use was independently associated with significantly lower intraoperative morphine consumption compared to LA. Surgery duration and bilateral hernia status were not significant predictors. Data are presented as mean ± SD. Statistical comparisons were performed using multiple linear regression, with t-tests used to determine the significance of individual regression coefficients. A p-value < 0.05 was considered statistically significant. RSB vs. LA: β = −3.53 mg, t = −6.26, 95% CI: −4.63 to −2.42, p < 0.0001. Surgery duration (per minute): β not statistically significant (p > 0.05). Bilateral hernia: β not statistically significant (p > 0.05).

**Figure 3:**
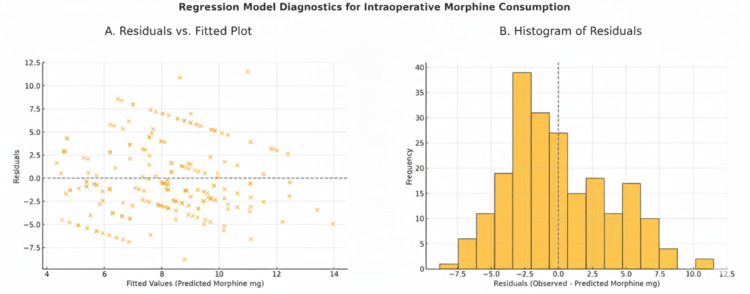
Regression Diagnostics for the Intraoperative Morphine Consumption Model in Rectus Sheath Block (RSB) and the Local Anesthetic (LA) Infiltration Groups. (A) Residuals vs. Fitted Plot: This plot visualizes residuals against the predicted intraoperative morphine equivalent values (mg) from the multivariable regression model. A random scatter around zero is expected in a well-fitted model. The mild funnel-shaped dispersion suggests potential heteroscedasticity. (B) Histogram of Residuals: This histogram displays the distribution of residuals (observed minus predicted morphine values). The distribution is moderately skewed but approximates normality, indicating reasonable fit for the assumptions of linear regression.

To account for potential confounding factors, a multiple linear regression analysis was performed with intraoperative morphine consumption as the dependent variable, and analgesic group, surgery duration, and hernia laterality as independent variables. The analysis revealed that the use of RSB was associated with a statistically significant reduction in morphine consumption Regression Coefficient (β) = −3.53 mg; 95% Confidence Interval (95% CI): −4.63 to −2.42; p-value (p) < 0.0001. Neither the duration of surgery nor the presence of bilateral hernia significantly impacted opioid requirements in this adjusted model. Model assumptions for the multiple linear regression analysis were assessed using visual inspection of residual plots, including a histogram of residuals. The residuals demonstrated an approximately normal distribution with no significant skewness or outliers, confirming the appropriateness of the linear regression approach (Figures 2, 3). These findings indicate that the application of RSB significantly reduces intraoperative opioid consumption in patients undergoing laparoscopic inguinal hernia repair, independent of surgery duration and hernia laterality.

Intraoperative dexmedetomidine requirements

**Figure 4:**
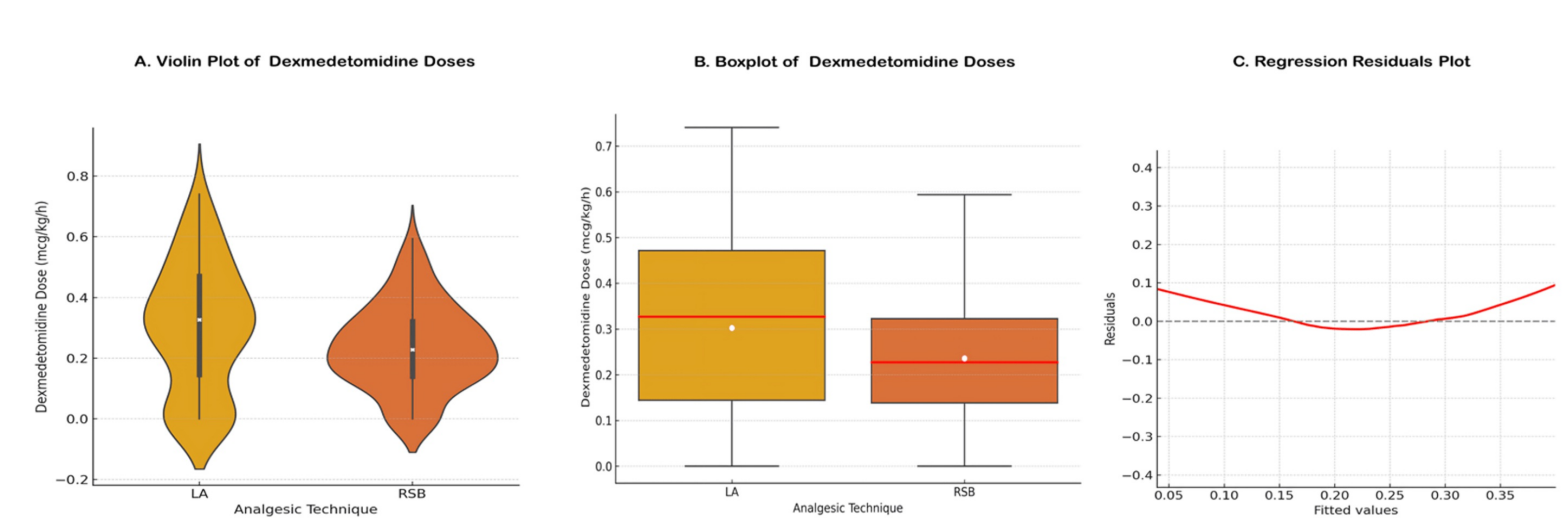
Dexmedetomidine Dose Comparison Between Analgesic Techniques. Panel A shows violin plots demonstrating the distribution and density of intraoperative dexmedetomidine infusion rates in the local anesthetic (LA) infiltration group (n = 109) and the rectus sheath block (RSB) group (n = 102). Panel B presents boxplots illustrating central tendency and variability, where the white dot in the violin plots and the black horizontal line in the boxplots both represent the median dexmedetomidine dose for each group. Panel C displays a residuals-versus-fitted-values plot, confirming an approximately random distribution of residuals and no significant violation of model assumptions. Data are represented as median (interquartile range (IQR)). The LA group demonstrated a median dose of 0.327 mcg/kg/h (IQR: 0.327), while the RSB group demonstrated a median dose of 0.227 mcg/kg/h (IQR: 0.184). Group comparison was performed using the Mann–Whitney U test, which demonstrated a statistically significant reduction in intraoperative dexmedetomidine requirements in the RSB group compared to the LA group (U = 4361.0, p = 0.0068). Statistical significance was defined as p < 0.05.

A total of 211 adult patients who underwent laparoscopic inguinal hernia repair were included in the final analysis. Of these, 102 patients received an RSB administered prior to surgical incision, while 109 patients received LA infiltration at the conclusion of the procedure. The median intraoperative dexmedetomidine infusion rate was significantly lower in the RSB group compared to the LA group: 0.227 mcg/kg/h (interquartile range [IQR]: 0.184) versus 0.327 mcg/kg/h (IQR: 0.327), respectively. This difference was statistically significant (Mann-Whitney U test: U = 4361.0, p = 0.0068), indicating that patients who received RSB required significantly less intraoperative dexmedetomidine than those who received LA infiltration (Figure 4). To adjust for potential confounding variables, including surgical duration and hernia laterality (unilateral vs bilateral), a multiple linear regression model was constructed. The model revealed the following (Table 2).

**Table 2:** Multivariable Linear Regression Analysis of Factors Associated With Intraoperative Dexmedetomidine Infusion Rate. This table presents the results of a multivariable linear regression model evaluating the association between analgesic technique (rectus sheath block (RSB) vs. local anesthetic (LA) infiltration), operative duration, and hernia laterality with intraoperative dexmedetomidine infusion dose (mcg/kg/h). Regression coefficients (β), standard errors, 95% confidence intervals (CI), and p-values are reported. A negative coefficient indicates a reduction in dexmedetomidine dose per unit increase in the predictor variable. RSB = rectus sheath block; LA = local anesthetic infiltration. A p-value < 0.05 was considered statistically significant.

Variable	Coefficient (β)	Standard Error (SE)	t-value	95% CI	p-value	Statistical Test
Intercept	0.511	0.036	14.19	0.440 to 0.582	<0.001	Linear regression (t-test)
RSB vs. LA (ref)	−0.031	0.024	−1.29	−0.078 to 0.016	0.194	Linear regression (t-test)
Duration (minutes)	−0.0025	0.0004	−6.25	−0.003 to −0.002	<0.001	Linear regression (t-test)
Bilateral hernia	0.007	0.025	0.28	−0.042 to 0.057	0.775	Linear regression (t-test)

A multivariable linear regression analysis was performed to examine whether the choice of analgesic technique (RSB vs. LA infiltration) independently influenced the intraoperative dexmedetomidine infusion rate after adjusting for operative duration and hernia laterality. The use of RSB was associated with a non-significant trend toward lower dexmedetomidine dosing compared to LA infiltration (β = −0.031, 95% CI: −0.078 to 0.016, p = 0.194). In contrast, operative duration demonstrated a significant negative association with dexmedetomidine infusion rate (β = −0.0025 per minute, 95% CI: −0.003 to −0.002, p < 0.001), indicating that longer cases were associated with slightly lower average infusion rates. Hernia laterality (bilateral vs. unilateral) was not associated with dexmedetomidine requirements (β = 0.007, 95% CI: −0.042 to 0.057, p = 0.775).

These findings indicate that after adjustment for case duration and laterality, RSB did not independently reduce dexmedetomidine infusion rate, despite the significant reduction observed in the unadjusted comparison between groups. The observed difference in dexmedetomidine dosing therefore appears to be influenced in part by operative duration.

Postoperative opioid requirements

**Figure 5:**
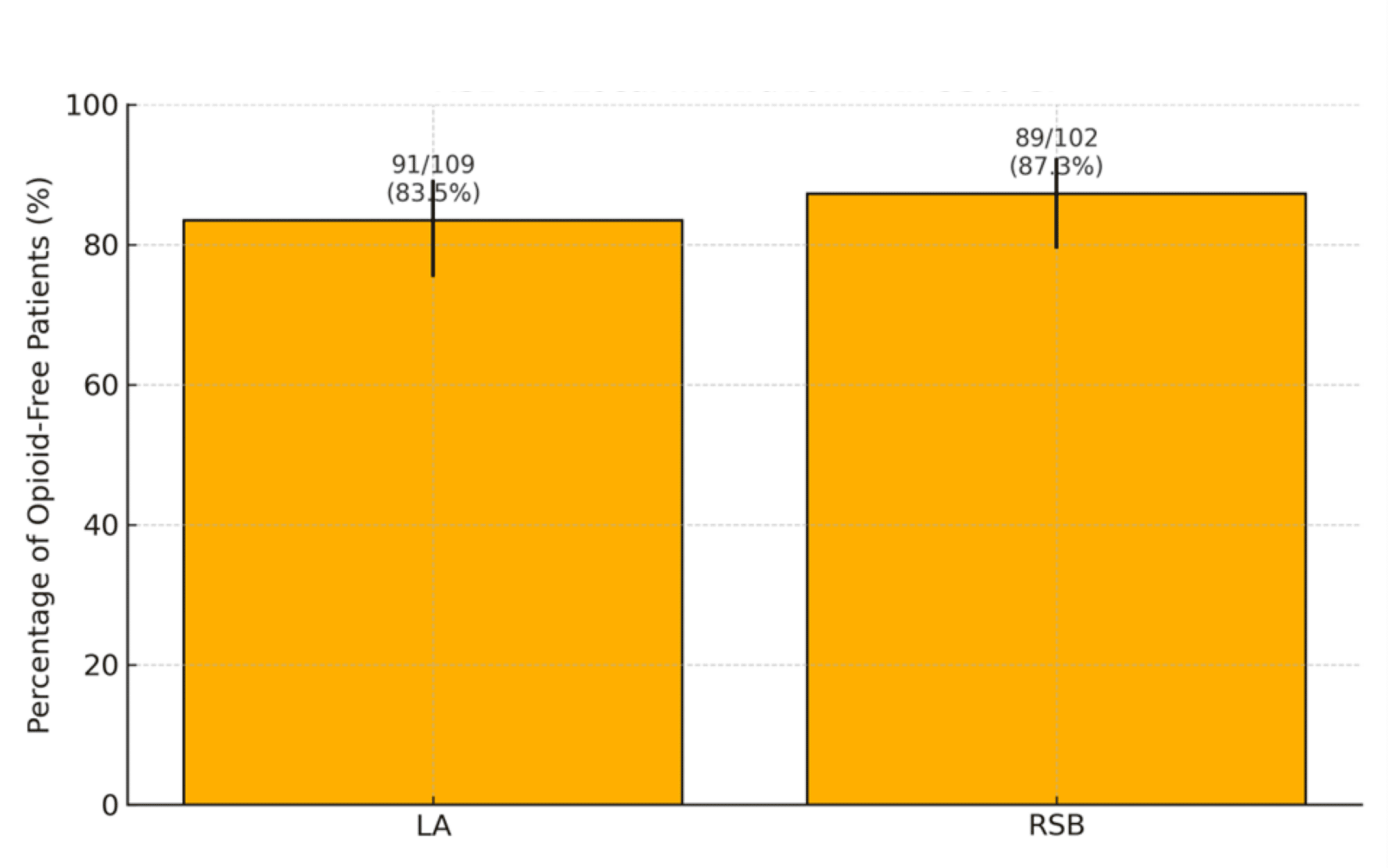
Percentage of Patients Not Requiring Postoperative Opioids in Rectus Sheath Block (RSB) and the Local Anesthetic (LA) Infiltration Groups. Bar graph illustrating the proportion of patients who did not require postoperative opioid analgesia in the Local Anesthetic (LA) group compared with the Rectus Sheath Block (RSB) group. A total of 91/109 patients (83.5%) in the LA group and 89/102 patients (87.3%) in the RSB group did not require postoperative opioids. A Chi-square test was used to compare the frequency of opioid-free recovery between groups, demonstrating no statistically significant difference (χ² = 0.33, p = 0.56).

A total of 211 patients were included: 102 received RSB and 109 received LA infiltration. The proportion of opioid-free patients was 87.3% in the RSB group and 83.5% in the LA group (Chi-square = 0.334, p = 0.563) (Figure 5).

Postoperative morphine consumption was minimal in both groups, with a median dose of 0.00 mg in both the LA infiltration and RSB groups. The mean morphine dose was slightly lower in the RSB group (1.17 ± 3.71 mg) compared to the LA group (1.35 ± 3.45 mg), although this difference was not statistically significant (p = 0.712, Mann-Whitney U test). The interquartile range (Q1-Q3) was 0.00-0.00 mg in both groups, indicating that the majority of patients in each group did not require any postoperative morphine. These findings suggest that both analgesic techniques were effective in minimizing postoperative opioid requirements (Table 3).

**Table 3:** Postoperative Morphine Consumption in the Rectus Sheath Block (RSB) and Local Anesthetic (LA) Infiltration Groups. Data are expressed as mean ± standard deviation (SD), median, range (Min–Max), and interquartile range (Q1–Q3). Postoperative morphine consumption between the local anesthetic (LA) infiltration and rectus sheath block (RSB) groups was compared using the Mann–Whitney U test. A two-tailed p-value < 0.05 was considered statistically significant. The difference between groups was not statistically significant (Z = 0.37, p = 0.712).

Group	Count	Mean (mg)	Median (mg)	SD	Min	Max	Q1–Q3 (mg)	p-value	Test Used
LA	109	1.35	0.00	3.45	0.0	20.0	0.00 – 0.00		
RSB	102	1.17	0.00	3.71	0.0	20.0	0.00 – 0.00	0.712	Mann–Whitney U

A multivariable logistic regression model was performed to assess whether the type of analgesic technique (RSB vs. local anesthetic infiltration), surgery duration, and hernia laterality were independently associated with postoperative opioid use. The analysis demonstrated no statistically significant association between the use of RSB and postoperative opioid requirement (β = −0.105, OR = 0.90, 95% CI 0.39-2.09, p = 0.806). Similarly, duration of surgery (β = −0.006, OR = 0.99, 95% CI 0.98-1.01, p = 0.320) and bilateral hernia repair (β = −0.512, OR = 0.60, 95% CI 0.25-1.43, p = 0.250) were not significantly associated with postoperative opioid use. These findings indicate that, after adjustment for operative duration and laterality, the application of RSB did not significantly influence the odds of requiring postoperative opioid analgesia in this cohort (Table 4).

**Table 4:** Multivariable Linear Regression Analysis of Factors Associated with Postoperative Opioid Requirements. Data represent logistic regression coefficients (β), standard errors (SE), z-values, and 95% confidence intervals (CI). Odds ratios (OR) were calculated as exp(β). The model examined predictors of being opioid-free postoperatively, including analgesic technique (rectus sheath block (RSB) vs. local anesthetic (LA)), duration of surgery (minutes), and hernia laterality (bilateral vs. unilateral). Statistical significance was evaluated using two-tailed z-tests, with p < 0.05 considered statistically significant.

Variable	Coefficient (β)	Std. Error	z-value	p-value	Odds Ratio (OR)	95% CI for OR	Model
Intercept	−0.8920	0.5086	−1.75	0.079	—	—	Logistic Regression
RSB vs. LA (ref)	−0.1054	0.4302	−0.25	0.806	0.90	0.39 – 2.09	Logistic Regression
Duration of Surgery (min)	−0.0061	0.0061	−0.99	0.320	0.99	0.98 – 1.01	Logistic Regression
Bilateral Hernia	−0.5118	0.4445	−1.15	0.250	0.60	0.25 – 1.43	Logistic Regression

Postoperative non-opioid analgesic requirements

**Figure 6:**
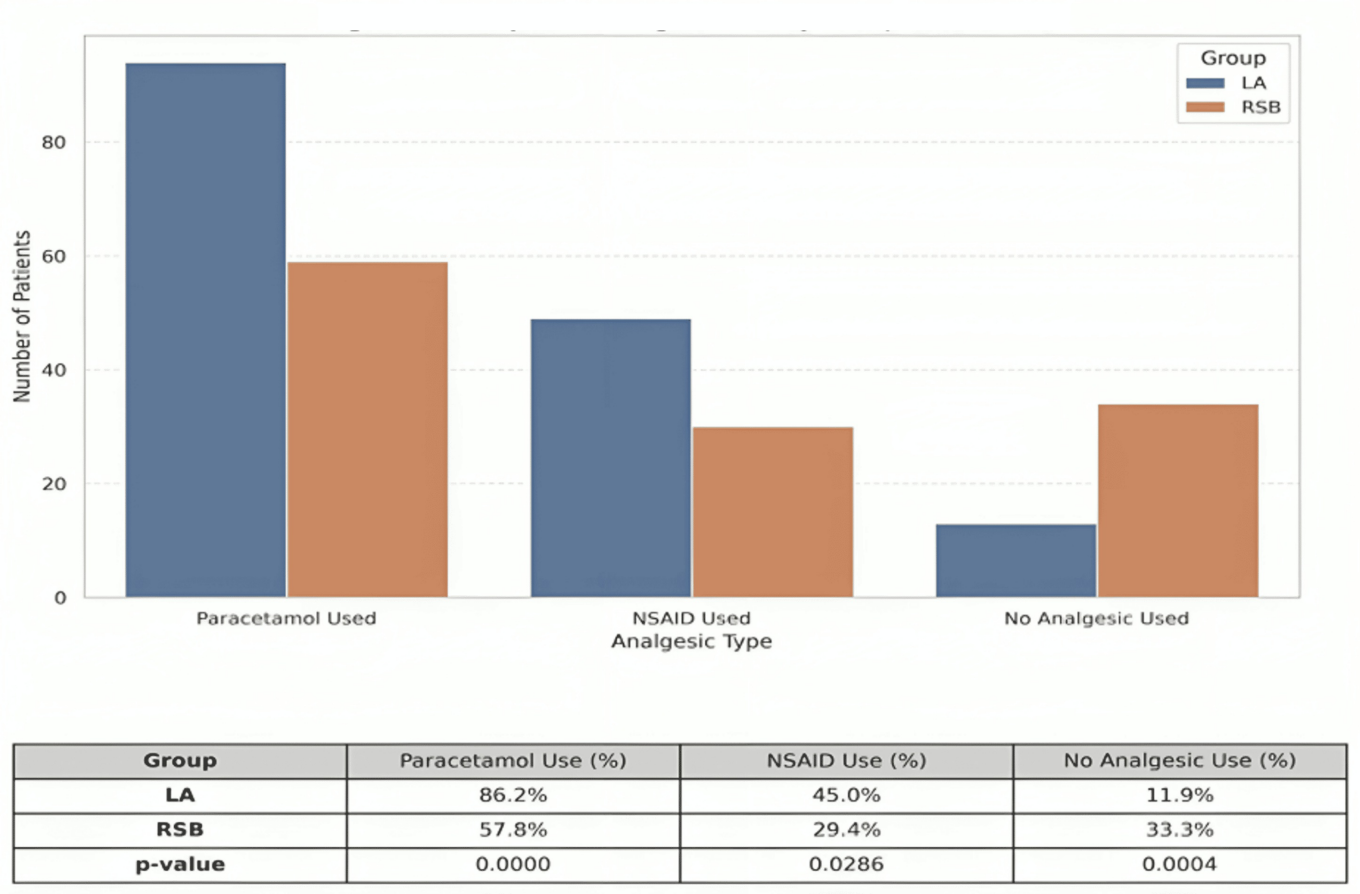
Unadjusted Postoperative Analgesic Use by Group Bar Chart Comparing Paracetamol, NSAID, and No Analgesia Use Between Rectus Sheath Block (RSB) Versus Local Anesthetic (LA) Infiltration Groups. Bar chart showing the number of patients who received each analgesic type postoperatively in the local anesthetic (LA) infiltration and rectus sheath block (RSB) groups. The accompanying table presents both the number of patients (N) and percentages (%) using each analgesic category. Data are expressed as N (%). Statistical comparisons between groups were performed using the chi-square (χ²) test. Paracetamol use: χ² = 21.38, p < 0.0001 NSAID use: χ² = 4.79, p = 0.0286 No analgesic use: χ² = 12.33, p = 0.0004 A p-value < 0.05 was considered statistically significant.

In the unadjusted analysis, patients in the RSB group demonstrated significantly lower postoperative analgesic requirements compared to those in the LA group. Paracetamol use was significantly more frequent in the LA group (86.2%) than in the RSB group (57.8%) (p < 0.0001). Similarly, NSAID use was higher in the LA group (45.0%) compared to the RSB group (29.4%) (p = 0.0286). In contrast, a significantly greater proportion of patients in the RSB group did not require any postoperative analgesia (33.3% vs. 11.9%, p = 0.0004), as shown in Figure 6.

Adjusted analysis of non-opioid analgesic use multivariable logistic regression was performed to assess the association between the analgesic technique and postoperative non-opioid analgesic use, adjusting for potential confounders including surgical duration and hernia laterality (Table 5).

**Table 5:** Adjusted Odds Ratios for Non-Opioid Analgesic Use between Rectus Sheath Block (RSB) versus Local Anesthetic (LA) Infiltration Groups. Data are expressed as odds ratios (OR) with 95% confidence intervals (CI), z-values, and p-values obtained from binary logistic regression analyses. Each model was adjusted for surgical laterality (unilateral vs bilateral) and duration of surgery (minutes). Statistical significance was set at p < 0.05.

Outcome	Variable	OR (95% CI)	z-value	p-value	Interpretation
Paracetamol Use	RSB vs LA	0.26 (0.13 – 0.51)	−3.84	<0.0001	Significantly lower odds with RSB
	Unilateral vs Bilateral	1.77 (0.84 – 3.71)	1.51	0.1325	Not significant
	Surgery Duration (min)	1.00 (0.99 – 1.01)	0.71	0.4801	Not significant
NSAID Use	RSB vs LA	0.54 (0.30 – 0.97)	−2.07	0.0389	Significantly lower odds with RSB
	Unilateral vs Bilateral	0.79 (0.42 – 1.48)	−0.74	0.4618	Not significant
	Surgery Duration (min)	0.99 (0.98 – 1.00)	−1.35	0.1772	Not significant
No Analgesic Use	RSB vs LA	3.03 (1.45 – 6.30)	2.95	0.0031	Significantly higher odds with RSB
	Unilateral vs Bilateral	0.63 (0.29 – 1.40)	−1.13	0.2602	Not significant
	Surgery Duration (min)	1.01 (1.00 – 1.02)	1.50	0.1331	Not significant

**Figure 7:**
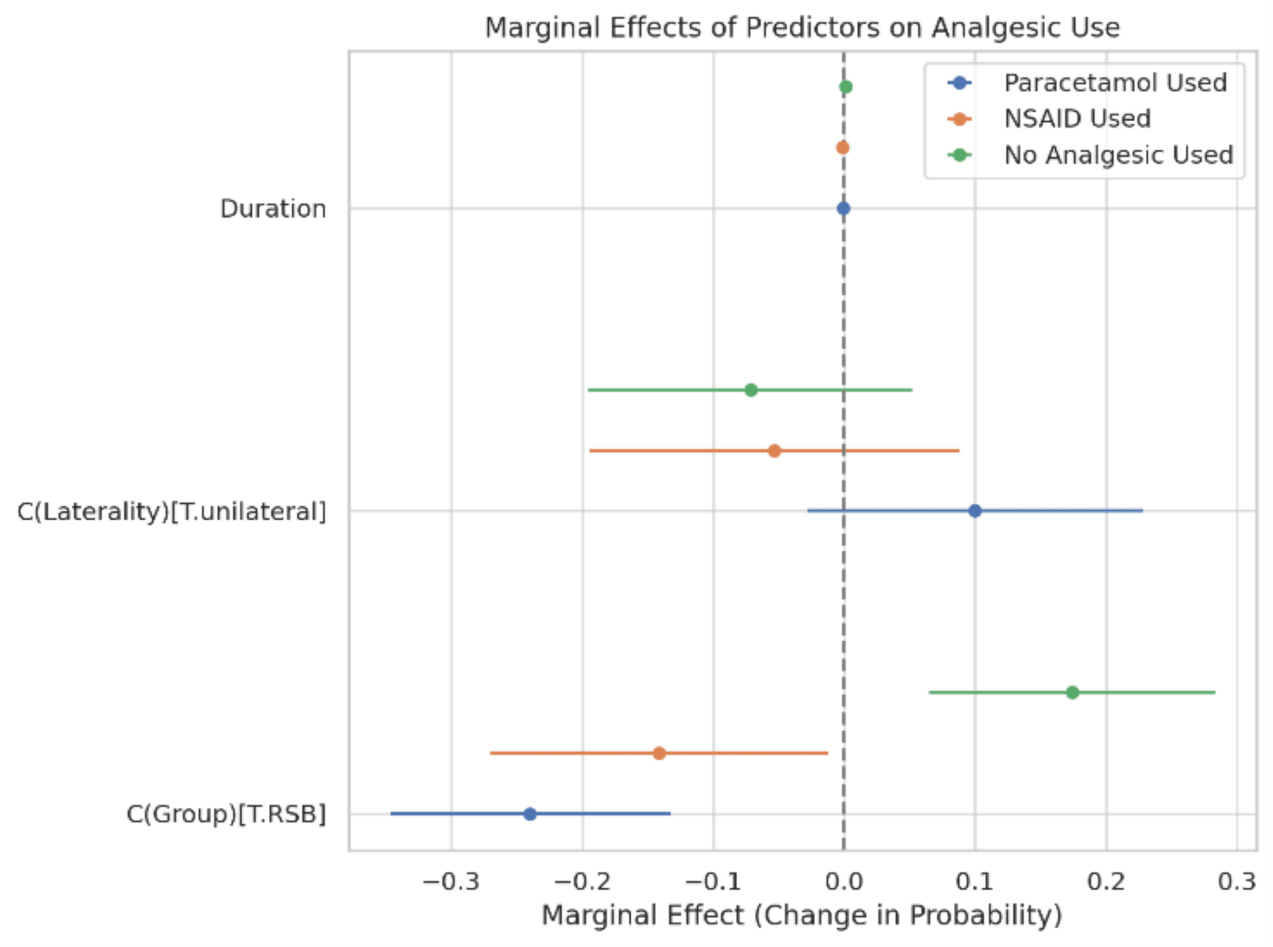
Marginal Effects of Predictors on Analgesic Use Marginal effects plot showing the change in predicted probability of receiving paracetamol, NSAIDs, or no analgesia after surgery, based on the multivariable logistic regression model. The model included analgesic technique (RSB vs. LA), hernia laterality (unilateral vs. bilateral), and duration of surgery (minutes) as predictors. Patients who received Rectus Sheath Block (RSB) had a lower predicted probability of receiving paracetamol and NSAIDs and a higher predicted probability of requiring no postoperative analgesics, compared to those who received Local Anesthetic infiltration. Surgical laterality and duration of surgery showed no clinically meaningful independent effects, with confidence intervals crossing zero. Data are presented as marginal effects (change in probability) with 95% confidence intervals, derived from the fitted regression model. A p-value < 0.05 was considered statistically significant. RSB: Rectus Sheath Block; LA: Local Anesthetic Infiltration; NSAID: Non-Steroidal Anti-Inflammatory Drug.

Figure 7 displays the adjusted odds ratios (AORs) with 95% confidence intervals for each predictor across analgesic categories. Compared to the LA group, patients who received RSB had significantly lower odds of receiving paracetamol (AOR: 0.26, 95% CI: 0.13-0.51) and NSAIDs (AOR: 0.54, 95% CI: 0.30-0.97), while the odds of receiving no analgesics were higher (AOR: 2.27, 95% CI: 1.12-4.58). Surgical laterality and duration did not significantly influence paracetamol or NSAID use but were weakly associated with the likelihood of receiving no analgesia, although the confidence intervals crossed unity.

**Figure 8:**
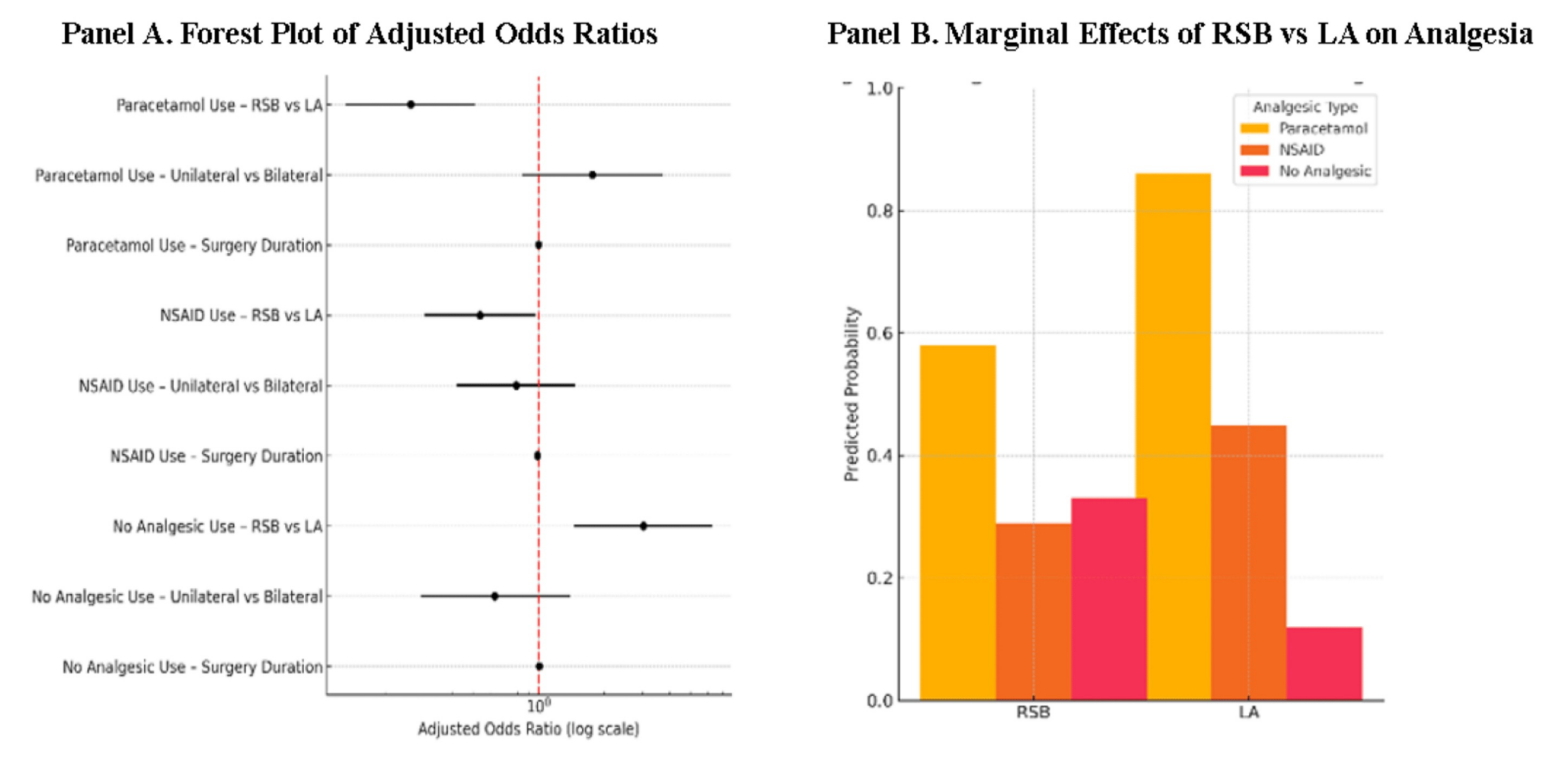
Adjusted Odds Ratios and Predicted Probabilities of Postoperative Non-opioid Analgesic Use in Rectus Sheath Block (RSB) versus Local Anesthetic (LA) Infiltration Groups. Panel A: Forest plot showing adjusted odds ratios (OR, 95% confidence intervals) derived from a multinomial logistic regression model evaluating independent predictors of postoperative Paracetamol, NSAID, or no analgesic use. The reference category for each comparison is local anesthetic (LA) infiltration. Panel B: Marginal effects plot showing predicted probabilities of each analgesic outcome by analgesic group (RSB vs LA), adjusted for hernia laterality and surgery duration. Data are expressed as OR (95% CI) for categorical predictors and marginal predicted probabilities (Mean ± SE) for continuous covariates. Statistical significance was defined at p < 0.05 (two-tailed). Model coefficients were estimated using the multinomial logistic regression test (z-statistic). Significant test statistic values from the model were as follows: RSB vs LA (Paracetamol use): z = −3.84, p < 0.0001 RSB vs LA (NSAID use): z = −2.07, p = 0.0389 RSB vs LA (No analgesic use): z = 2.95, p = 0.0031 All other predictors (laterality, duration) were not statistically significant (p > 0.05). RSB: Rectus Sheath Block; LA: Local Anesthetic; NSAID: Non-steroidal Anti-inflammatory Drug.

The marginal effects plot Figure 8 (Panel A) further illustrates the adjusted change in predicted probability of each analgesic type in relation to each predictor. Receiving RSB, compared to LA, was associated with a 32% absolute reduction in the probability of paracetamol use and an 11% reduction in NSAID use, with a corresponding 21% increase in the probability of receiving no analgesia. Laterality (unilateral surgery) had minimal influence on paracetamol or NSAID use but slightly increased the likelihood of no analgesia. Operative duration had a negligible marginal effect on all analgesic categories.

Figure 8 (Panel B) visualizes the predicted probabilities derived from the model, showing that the likelihood of receiving paracetamol was markedly higher in the LA group (~90%) compared to the RSB group (~58%), while the predicted use of NSAIDs was moderately reduced with RSB. Notably, the probability of receiving no postoperative analgesia was substantially higher in the RSB group (~28%) than in the LA group (~8%). These findings indicate that the use of RSB is independently associated with a reduced need for postoperative systemic analgesics, supporting its potential role as an effective opioid-sparing technique in laparoscopic inguinal hernia repair.

Frequency of postoperative non-opioid analgesic use

**Figure 9:**
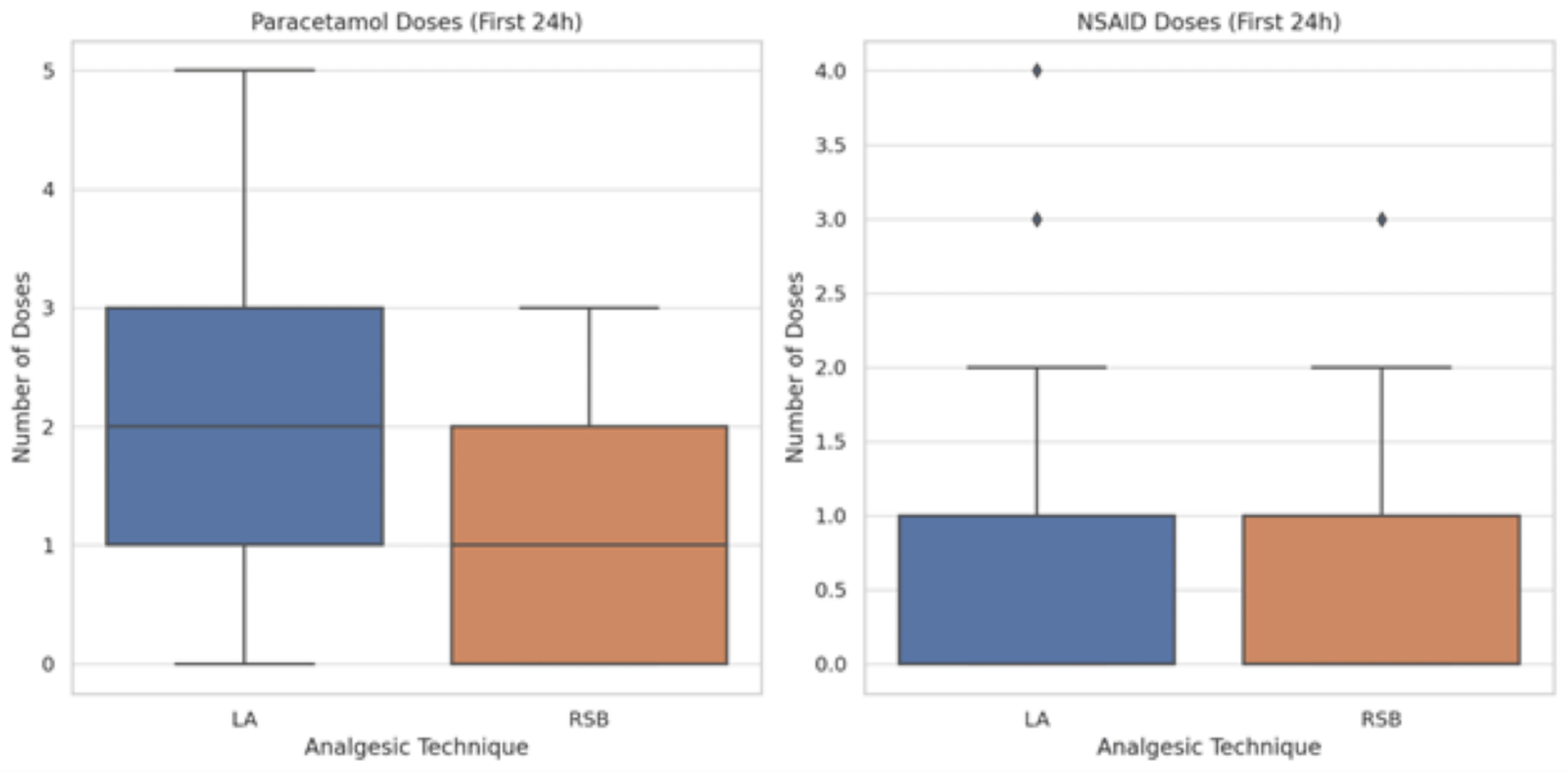
Postoperative Non-opioid Doses Within 24 H in Rectus Sheath Block (RSB) and Local Anesthetic (LA) Infiltration Groups. Panel A displays the number of paracetamol doses administered, and Panel B shows NSAID doses. Each boxplot illustrates the median, interquartile range (IQR), and full range of doses. Patients in the RSB group received significantly fewer doses of both paracetamol (median: 1 [IQR: 0–2] vs. 3 [IQR: 2–3], p < 0.001) and NSAIDs (median: 0 [IQR: 0–1] vs. 1 [IQR: 0–1], p = 0.027) compared to the LA group (Mann–Whitney U test). These results indicate reduced reliance on non-opioid analgesics with the use of RSB.  A p-value < 0.05 was considered statistically significant.

The frequency of non-opioid analgesic use within the first 24 hours postoperatively differed significantly between the RSB and LA groups. Patients in the RSB group required significantly fewer paracetamol doses compared to those in the LA group, with a median of 1 dose (interquartile range (IQR): 0-2) versus 3 doses (IQR: 2-3), respectively (p < 0.001, Mann-Whitney U test). Similarly, NSAID use was lower in the RSB group, with a median of 0 doses (IQR: 0-1) compared to 1 dose (IQR: 0-1) in the LA group (p = 0.027) (Figure 9). To adjust for potential confounding variables, a multivariable Poisson regression analysis was performed, incorporating surgery duration and hernia laterality. After adjustment, the RSB technique remained independently associated with a significant reduction in paracetamol dose requirements (incidence rate ratio [IRR] 0.65; 95% CI: 0.54-0.78; p < 0.001). NSAID use was also significantly lower in the RSB group after adjustment (IRR 0.77; 95% CI: 0.60-0.99; p = 0.043). These findings confirm that RSB is associated with reduced reliance on non-opioid analgesics during the early postoperative period (Table 6).

**Table 6:** Unadjusted and Adjusted Comparisons of Paracetamol and NSAID Doses Administered Within the First 24 Hours Postoperatively Between Rectus Sheath Block (RSB) and Local Anesthetic (LA) Infiltration Groups. Comparisons between groups (RSB vs LA) were performed using the Mann–Whitney U test for unadjusted analysis (reported as U-statistic and p-value) and a multivariable Poisson regression model for adjusted comparisons (expressed as incidence rate ratio (IRR) with 95% confidence interval (CI)). The test statistic values for the Mann–Whitney comparisons were: Paracetamol administrations: U = 3,196.5, p < 0.0001 NSAID administrations: U = 4,762.0, p = 0.027 The adjusted Poisson regression yielded: Paracetamol administrations: IRR = 0.53 (95% CI: 0.42–0.68), z = −4.35, p < 0.001 NSAID administrations: IRR = 0.65 (95% CI: 0.41–1.04), z = −1.80, p = 0.070 Statistical significance was defined at p < 0.05 and p < 0.001 as highly significant.

Outcome (administrations per patient, 0–24 h)	RSB, median (IQR)	LA, median (IQR)	Unadjusted p (Mann–Whitney U)	Adjusted IRR (95% CI)	Adjusted p
Paracetamol administrations	1 (0–2)	2 (1–3)	<0.0001	0.53 (0.42–0.68)	<0.001
NSAID administrations	0 (0–1)	0 (0–1)	0.027	0.65 (0.41–1.04)	0.070

Postoperative analgesic outcomes between LA and RSB groups

**Figure 10:**
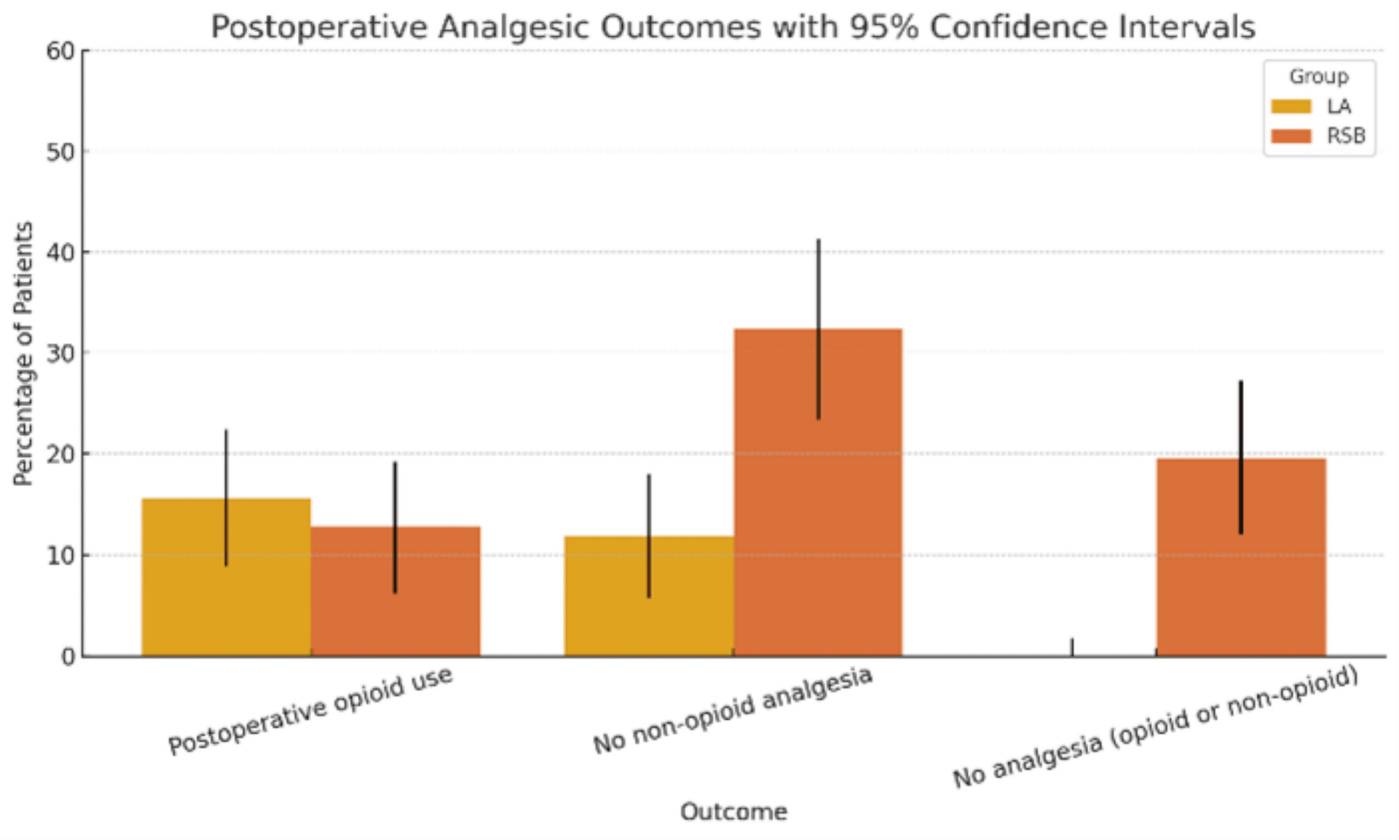
Comparison of Postoperative Analgesic Outcomes Between Rectus Sheath Block (RSB) and Local Anesthetic (LA) Infiltration Groups. Bar heights represent the proportion of patients (%) experiencing each outcome, with 95% confidence intervals shown. The RSB group demonstrated lower opioid use, higher rates of non-opioid sparing, and a substantial percentage of patients requiring no postoperative analgesia.

Postoperative analgesic outcomes are summarized in Table 7 and Figure 10. The proportion of patients requiring postoperative opioids was slightly lower in the RSB group (12.7%) compared to the LA group (15.6%), though this difference was not statistically significant (Risk Ratio (RR) = 0.82, 95% CI: 0.42-1.60, p = 0.6925). In contrast, the proportion of patients who did not receive postoperative non-opioid analgesics (paracetamol or NSAIDs) was significantly higher in the RSB group (32.4%) than in the LA group (11.9%), with a relative risk of 2.71 (95% CI: 1.52-4.86, p = 0.0006). Most notably, 20 patients (19.6%) in the RSB group required no postoperative analgesia at all, compared to 0 patients (0.0%) in the LA group. This difference was statistically significant (RR = 43.79, 95% CI: 2.68-714.71, p < 0.0001), suggesting a clinically meaningful impact of RSB in eliminating the need for both opioid and non-opioid postoperative analgesia in a substantial proportion of patients.

**Table 7:** Comparison of Postoperative Analgesic Outcomes Between Rectus Sheath Block (RSB) and Local Anesthetic (LA) Infiltration Groups. Data are presented as n/N (%) for categorical variables. Between-group comparisons were conducted using the Chi-square (χ²) test, and results are expressed as risk ratios (RR) with 95% confidence intervals (CI). The corresponding χ² test statistics were: Postoperative opioid use: χ² = 0.16, p = 0.6925 No postoperative non-opioid analgesia: χ² = 11.76, p = 0.0006 No postoperative analgesia (opioid or non-opioid): χ² = 19.09, p < 0.0001 Statistical significance was defined at p < 0.05, and values of p < 0.001 were considered highly significant.

Outcome	LA: n/N (%)	RSB: n/N (%)	RR	95% CI	Test	p-value
Postoperative opioid use	17 / 109 (15.6%)	13 / 102 (12.7%)	0.82	[0.42 – 1.60]	Chi-square	0.6925
No postoperative non-opioid analgesia	13 / 109 (11.9%)	33 / 102 (32.4%)	2.71	[1.52 – 4.86]	Chi-square	0.0006
No postoperative analgesia (opioid or non-opioid)	0 / 109 (0.0%)	20 / 102 (19.6%)	43.79	[2.68 – 714.71]	Chi-square	< 0.0001

**Figure 11:**
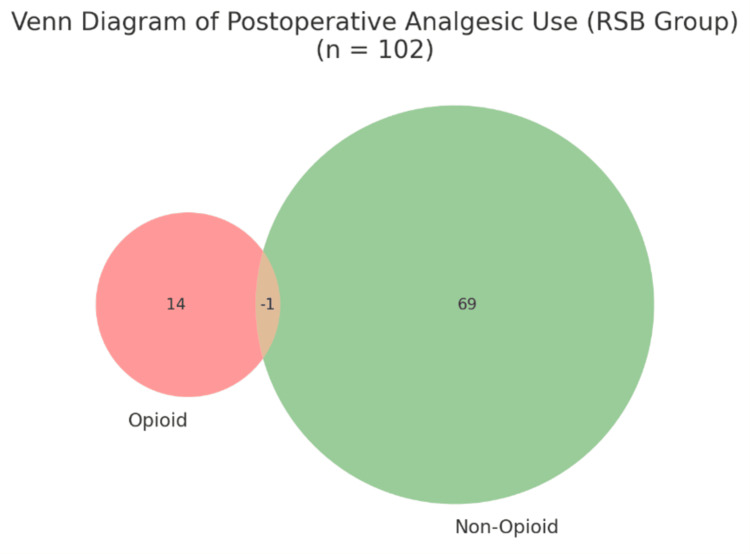
Venn Diagram Illustrating Postoperative Analgesic Use Patterns in the Rectus Sheath Block (RSB) Group. This diagram illustrates the overlap of postoperative opioid and non-opioid analgesic use in patients who received rectus sheath block (RSB). Notably, 20 patients (19.6%) required neither opioids nor non-opioid analgesics postoperatively, indicating complete analgesia freedom following RSB. The figure above visually represents the distribution of postoperative analgesic use in the RSB group (n = 102): Left circle: Patients who received opioids Right circle: Patients who received non-opioid analgesics Overlap: Patients who received both Outside both circles: 20 patients who required no analgesia at all

The RSB group had a significantly higher likelihood of avoiding both opioid and non-opioid use compared to LA. The difference in opioid avoidance alone was not statistically significant (p = 0.6925). Both non-opioid sparing and complete analgesia-free recovery outcomes were significantly better in RSB (Figure 10, Figure 11).

## Discussion

This retrospective study evaluated the clinical impact of RSB administered preoperatively versus LA infiltration provided at the end of surgery, as components of multimodal analgesia for laparoscopic inguinal hernia repair. Our findings support the hypothesis that RSB, when performed before incision, offers superior intraoperative analgesic benefits and reduces the need for postoperative non-opioid medications, without compromising postoperative opioid control.

Although demographic characteristics were comparable between groups, procedural differences were noted: the RSB group had significantly longer operative times (100.2 ± 31.4 minutes vs. 85.3 ± 28.8 minutes; p < 0.001) and a higher incidence of bilateral hernia repairs (70.6% vs. 48.6%; p = 0.002). These differences likely reflect clinical decision-making, where regional techniques like RSB are preferentially used for more extensive or bilateral procedures due to anticipated higher pain burden. As such, these baseline imbalances may introduce confounding if unaddressed. To mitigate this, we incorporated surgical duration and laterality as covariates in all adjusted analyses. This approach ensures a more accurate estimation of the independent analgesic effect of RSB and highlights the importance of contextualizing procedural complexity in retrospective studies.

Intraoperative opioid and dexmedetomidine consumption

The present study demonstrated that RSB significantly reduced intraoperative opioid consumption compared to LA infiltration in adult patients undergoing laparoscopic inguinal hernia repair using the TEP technique. Patients in the RSB group required substantially lower mean intraoperative morphine-equivalent doses (6.65 mg vs. 9.50 mg; p < 0.0001). After adjusting for operative duration and hernia laterality, multivariable linear regression confirmed RSB as an independent predictor of reduced opioid requirement (β = −3.53 mg; 95% CI: −4.63 to −2.42; p < 0.0001). Model diagnostics indicated a good fit, with residuals exhibiting near-normal distribution and minimal heteroscedasticity.

These findings underscore the intraoperative analgesic efficacy of RSB and its potential contribution to enhanced perioperative opioid stewardship. While previous randomized data in pediatric populations have similarly shown that RSB reduces intraoperative opioid use during laparoscopic inguinal hernia repair (mean 0.115 mg/kg vs. 0.144 mg/kg; p < 0.001) [1]. Our results align with prior reports that abdominal wall nerve blocks can reduce intraoperative opioid requirements. For example, Kim et al. observed that the use of rectus sheath and quadratus lumborum blocks halved remifentanil consumption in laparoscopic hernia surgery protocols [9]. Similarly, Shi et al. reported that incision-based RSB significantly reduced opioid usage in laparoscopic abdominal operations [10]. Thus, our findings extend the evidence base by highlighting the benefit of RSB in the intraoperative rather than just the postoperative period, and in the specific context of laparoscopic inguinal hernia repair.

However, most existing RSB studies focus on postoperative outcomes (pain scores, 24-h opioid consumption), not intraoperative dosing. Thus, our study adds new evidence that the block can modify analgesic demand even during the active surgical period. The reduced intraoperative opioid use in the RSB group can be attributed to its mechanism of blocking somatic pain signals from the anterior abdominal wall. By anesthetizing the terminal branches of the thoracoabdominal nerves (T7-T12), RSB minimizes nociceptive input during trocar insertion and abdominal wall manipulation, leading to less central pain transmission and lower opioid requirements to maintain hemodynamic stability. However, as RSB mainly targets somatic rather than visceral pain, some opioid use may still be needed for visceral stimuli such as peritoneal traction or insufflation [1].

This study assessed the effect of RSB on intraoperative dexmedetomidine infusion rates in adults undergoing laparoscopic inguinal hernia repair. In the unadjusted analysis, patients who received RSB required a significantly lower median dexmedetomidine infusion rate compared with those who received LA infiltration at the end of surgery (0.227 vs. 0.327 mcg/kg/h; p = 0.0068). This finding suggests that RSB provided effective pre-emptive analgesia, reducing nociceptive input and blunting the intraoperative stress response, which in turn decreased the need for sedative agents such as dexmedetomidine.

After adjustment for surgical duration and hernia laterality-both significantly different between the two groups-the association between RSB and reduced dexmedetomidine requirement was no longer statistically significant (adjusted p = 0.194). This indicates that the initial difference was likely influenced by confounding factors, as patients in the RSB group more often underwent longer and bilateral procedures, which can independently affect sedative use. In the multivariable model, operative duration remained a significant independent predictor of dexmedetomidine infusion rate (β = -0.0025; p < 0.001), showing a paradoxical decrease in infusion rate with longer operative time. This trend may reflect intraoperative titration protocols aimed at maintaining hemodynamic stability during prolonged procedures, or a ceiling effect from the effective pre-emptive analgesia provided by RSB that limited the need for additional sedation.

Although the adjusted analysis did not demonstrate a statistically significant association, the clinical implications remain relevant. The modest effect size (β = -0.031) suggests that RSB contributes to a smoother and more stable anesthetic course, even if its direct influence on sedative requirements is limited when other perioperative variables are considered. These findings emphasize the importance of controlling for confounders in retrospective analyses and support the inclusion of RSB as a valuable component of multimodal analgesia strategies in laparoscopic hernia repair.

Physiologically, RSB attenuates afferent nociceptive transmission from the anterior abdominal wall, leading to reduced sympathetic activation and a lower requirement for α2-agonist supplementation to maintain stable hemodynamics [11]. Similar intraoperative sedative-sparing effects of regional blocks have been demonstrated with TAP and paravertebral blocks in various laparoscopic procedures [12,13]. Nahla et al. also observed that the addition of dexmedetomidine to local anesthetics in abdominal wall blocks enhanced intraoperative hemodynamic stability and prolonged postoperative analgesia in pelvic-abdominal surgery [14]. Although their focus was on block adjuvants rather than systemic infusion, the pharmacologic rationale supports the present findings.

Collectively, these results suggest that RSB contributes to decreased intraoperative sedative and analgesic demands through superior somatic analgesia and attenuation of stress responses. The lack of statistical significance after covariate adjustment underscores that while RSB has an independent sparing trend, its magnitude is modulated by surgical duration and procedure laterality

Postoperative outcomes

Although VAS scores were recorded, they were often documented after analgesic administration, potentially reflecting post-treatment rather than baseline pain levels. Therefore, analgesic use was employed as a surrogate marker of pain intensity. Given that our institution follows a standardized protocol reserving opioids for severe pain (VAS >7) and administering paracetamol or NSAIDs for mild to moderate pain (VAS 1-6) the type and frequency of medication provided a consistent, objective measure for comparing postoperative pain management between groups.

Effect of RSB on postoperative opioid use

In this retrospective comparative study, we found no statistically significant difference in postoperative opioid use between patients who received RSB prior to laparoscopic inguinal hernia repair and those who received LA infiltration at the end of surgery. The majority of patients in both groups remained opioid-free in the immediate postoperative period, with 87.3% of RSB patients and 83.5% of LA patients not requiring any postoperative opioids (p = 0.563). Median morphine-equivalent consumption was 0.00 mg in both groups, and adjusted regression models confirmed no significant association between analgesic technique and postoperative opioid requirements (adjusted β = -0.1054, 95% CI: -0.9486 to 0.7378, p = 0.806).

These findings suggest that both RSB and LA infiltration are similarly effective in controlling postoperative pain to an extent that minimizes opioid need. Although previous literature has demonstrated the analgesic benefits of RSB in abdominal surgeries, including reduced opioid consumption in open procedures or when compared to no block at all [13], our data align with studies showing comparable outcomes between RSB and other local techniques in minimally invasive settings [15,16].

An important consideration in interpreting these results is the use of standardized multimodal analgesia across both groups in our study. All patients received paracetamol, NSAIDs, and intraoperative dexmedetomidine as part of a protocolized pain management strategy. Multimodal analgesia has been shown to be effective in minimizing opioid consumption by targeting different nociceptive pathways [17]. In this context, while we observed significantly reduced intraoperative opioid requirements in the RSB group, the additive benefit of RSB on postoperative opioid consumption may have been mitigated by the strong baseline analgesia provided to all patients.

Our regression analysis also confirmed that other procedural variables, such as operative duration and hernia laterality, did not significantly influence postoperative opioid use. This further supports the robustness of our findings across varying surgical complexities. Nonetheless, RSB may still offer advantages in specific contexts, such as in patients where longer or bilateral procedures are anticipated, as suggested by trends in previous studies [18,19]. However, our data did not demonstrate such subgroup effects, potentially due to the small number of patients who required any postoperative opioids.

In conclusion, in laparoscopic TEP inguinal hernia repair, both rectus sheath block and local anesthetic infiltration provide effective postoperative analgesia, with similarly low opioid requirements. In the context of standardized multimodal analgesia with paracetamol, NSAIDs, and dexmedetomidine, the incremental benefit of RSB in reducing postoperative opioid use appears limited, although its intraoperative opioid-sparing effect remains evident.

Postoperative non-opioid analgesic use

Our study demonstrates that the use of RSB in laparoscopic inguinal hernia repair is significantly associated with reduced postoperative non-opioid analgesic consumption compared to LA infiltration. Both unadjusted and adjusted analyses consistently revealed that patients who received RSB required fewer doses of paracetamol and NSAIDs and were more likely to require no additional postoperative analgesia.

In the unadjusted analysis, the proportion of patients requiring paracetamol and NSAIDs was significantly lower in the RSB group. Importantly, a third of patients in the RSB group required no postoperative analgesia at all, highlighting the potential of RSB to provide effective somatic pain control. These findings remained robust in the multivariable logistic regression analysis after adjustment for surgical duration and hernia laterality, suggesting that the analgesic benefit of RSB is independent of procedural complexity.

The adjusted odds ratio (AOR) for paracetamol use with RSB was 0.26 (95% CI: 0.13-0.51; p = 0.0001), and for NSAID use was 0.54 (95% CI: 0.30-0.97; p = 0.0389), indicating a significant reduction in the likelihood of requiring these agents. Additionally, the odds of receiving no postoperative analgesia were more than three times higher in the RSB group (AOR: 3.03; 95% CI: 1.45-6.30; p = 0.0031). Marginal effects analysis further supported these outcomes, showing that RSB reduced the predicted probability of paracetamol use by 32% and increased the likelihood of requiring no analgesics by 21%.

These results align with the mechanistic rationale that RSB effectively blocks somatic nociceptive input from the anterior abdominal wall, thereby attenuating postoperative pain and reducing systemic analgesic demand. Previous randomized controlled trials and observational studies have demonstrated similar findings. For instance, Teshome et al. found that RSB provided superior postoperative analgesia compared to standard opioid-based regimens following midline abdominal surgery, reducing the need for both opioids and non-opioid adjuncts [20]. Likewise, Ra et al. reported that patients undergoing laparoscopic procedures with preemptive RSB had significantly lower postoperative pain scores and reduced analgesic consumption [21].

The observed reduction in paracetamol and NSAID use with RSB is clinically significant, considering the potential adverse effects associated with systemic analgesics. NSAIDs, for instance, can lead to gastrointestinal irritation, renal dysfunction, and bleeding risk, particularly in surgical patients. Paracetamol, although generally safe, poses hepatotoxic risk in high or prolonged doses. Thus, the opioid- and non-opioid-sparing effects of RSB may contribute not only to better analgesia but also to enhanced patient safety and recovery.

Despite the significant differences in analgesic use, it is notable that surgical duration and laterality did not significantly affect postoperative non-opioid analgesic requirements in our adjusted models. This strengthens the inference that the analgesic efficacy of RSB is not merely a reflection of less complex surgeries being assigned to this technique, but rather a true physiological benefit.

The observed analgesic benefit of RSB can be explained by its anatomical spread pattern and the somatic nerves it targets. When performed via a lateral or posterolateral approach particularly near the lateral border of the rectus abdominis the injectate may extend beyond the posterior rectus sheath into adjacent fascial planes. Cadaveric and radiologic studies have demonstrated potential cephalocaudal spread from the T7 to L1 levels when sufficient injectate volume is used (≥20 mL per side) [22,23]. Owing to the continuity between the posterior rectus sheath and the neurofascial plane between the internal oblique and transversus abdominis muscles, local anesthetic can diffuse laterally and posteriorly to reach the L1 nerve branches [15]. Consequently, a lateral RSB approach may occasionally result in partial blockade of the iliohypogastric and ilioinguinal nerves, in addition to the thoracoabdominal intercostal nerves (T7-T12). This extended neural coverage explains why RSB can provide analgesia not only to the midline but also to the lateral and inguinal regions, which are relevant to laparoscopic hernia repair.

In the context of the present study, this anatomical mechanism may account for the significantly lower intraoperative dexmedetomidine requirements and reduced postoperative analgesic use observed in the RSB group compared with local infiltration. By potentially blocking both thoracoabdominal and L1 sensory afferents, RSB provides more effective attenuation of nociceptive input during trocar insertion and peritoneal traction, contributing to intraoperative hemodynamic stability and a reduced need for systemic sedatives and opioids.

In conclusion, the implementation of RSB as part of a multimodal analgesic strategy in laparoscopic inguinal hernia repair significantly reduces the need for postoperative paracetamol and NSAIDs and increases the likelihood of achieving analgesia without systemic agents. These results support the broader application of RSB as an effective, opioid-sparing regional technique in minimally invasive abdominal surgery.

Strengths and limitations

Strengths include the single-procedure adult cohort, standardized conversion of all opioids to IV morphine equivalents, and consistent findings across unadjusted and adjusted analyses. Although the RSB group had longer operative duration and more bilateral repairs, opioid sparing effects were still observed, reducing the likelihood of confounding by indication. Limitations include the retrospective, nonrandomized design and very low postoperative opioid event rates, which limit power to detect between group differences. Because the study compares a pre-incisional analgesic technique (RSB) with a postoperative local infiltration approach, inherent differences in timing may influence the interpretation of temporal analgesic effects. However, this reflects real-world clinical practice and the routinely performed sequence of each technique, which was intentionally preserved to maintain external validity. Future prospective studies could incorporate comparator regional techniques such as the TAP block to further contextualize the relative analgesic contribution of RSB in laparoscopic inguinal hernia repair.

## Conclusions

In this retrospective comparative study of adult patients undergoing laparoscopic inguinal hernia repair, the pre-incisional RSB was associated with a significant reduction in intraoperative opioid requirements compared with LA wound infiltration. Despite the RSB group having longer operative times and a higher proportion of bilateral hernia repairs, the association remained consistent after statistical adjustment, indicating a true analgesic advantage during the intraoperative phase.

Postoperative opioid requirements were minimal in both groups, likely reflecting multimodal pain management protocols and the generally low postoperative opioid demand in minimally invasive hernia surgery. However, patients who received RSB demonstrated lower use of non-opioid rescue analgesics and a higher proportion of complete analgesia avoidance, suggesting improved early postoperative comfort and reduced analgesic dependency. Overall, these findings support RSB as a beneficial component of perioperative analgesia in laparoscopic inguinal hernia repair, particularly for reducing intraoperative opioid exposure and enhancing postoperative analgesic efficiency. RSB may be considered as part of opioid-sparing and enhanced-recovery strategies in this surgical population.
